# High-frequency ventilation in preterm infants and neonates

**DOI:** 10.1038/s41390-021-01639-8

**Published:** 2022-02-08

**Authors:** Benjamin W. Ackermann, Daniel Klotz, Roland Hentschel, Ulrich H. Thome, Anton H. van Kaam

**Affiliations:** 1 Division of Neonatology, Department of Women’s and Children’s medicine, University Hospital Leipzig, Leipzig, Germany; 2 Center for Pediatrics, Department of Neonatology, Medical Center – University of Freiburg, Faculty of Medicine, University of Freiburg, Freiburg, Germany; 3 Division of Neonatology, Emma Children’s Hospital Amsterdam UMC, University of Amsterdam, Amsterdam, The Netherlands

## Abstract

**Abstract:**

High-frequency ventilation (HFV) has been used as a respiratory support mode for neonates for over 30 years. HFV is characterized by delivering tidal volumes close to or less than the anatomical dead space. Both animal and clinical studies have shown that HFV can effectively restore lung function, and potentially limit ventilator-induced lung injury, which is considered an important risk factor for developing bronchopulmonary dysplasia (BPD). Knowledge of how HFV works, how it influences cardiorespiratory physiology, and how to apply it in daily clinical practice has proven to be essential for its optimal and safe use. We will present important aspects of gas exchange, lung-protective concepts, clinical use, and possible adverse effects of HFV. We also discuss the study results on the use of HFV in respiratory distress syndrome in preterm infants and respiratory failure in term neonates.

**Impact:**

- Knowledge of how HFV works, how it influences cardiorespiratory physiology, and how to apply it in daily clinical practice has proven to be essential for its optimal and safe use.
- Therefore, we present important aspects of gas exchange, lung-protective concepts, clinical use, and possible adverse effects of HFV.
- The use of HFV in daily clinical practice in lung recruitment, determination of the optimal continuous distending pressure and frequency, and typical side effects of HFV are discussed.
- We also present study results on the use of HFV in respiratory distress syndrome in preterm infants and respiratory failure in term neonates.

## Introduction

High-frequency ventilation (HFV) is an exceptional invasive mechanical ventilation mode, in which gas transport and gas mixing are distinctly different from all other modes of mechanical ventilation.^1^ Developed by groups in Germany and Canada, HFV is characterized by the delivery of very small tidal volumes (*V*_T_) at supra-physiological frequencies.^2,3^ Animal studies have shown that ventilation with small *V*_T_, together with avoiding alveolar collapse, is one of the fundamental principles to minimize ventilator-induced lung injury (VILI).^4,5^ Preterm infants also benefitted from small tidal volumes compensated by higher ventilation rates.^6–10^ VILI is considered an important risk factor in the development of bronchopulmonary dysplasia (BPD).^11^ HFV has been considered a lung-protective ventilation modality since inspiratory overdistention due to large V_T_, and end-expiratory lung collapse due to insufficient airway pressures may be more efficiently avoided than in conventional mechanical ventilation (CMV). For these reasons, HFV has been frequently used in neonatal care over the last 30 years.^12,13^

Knowledge of how HFV works, how it impacts physiology, and how to apply it in daily clinical practice has proven to be essential for its optimal and safe use. This paper aims to review essential aspects of HFV and to summarize the available evidence for its therapeutic use in newborn infants.

## Basic principles

### Gas exchange during HFV

Although the goals for gas exchange and avoiding VILI are similar during HFV and CMV, the mechanisms to accomplish these goals are fundamentally different.

**Fig. 1:**
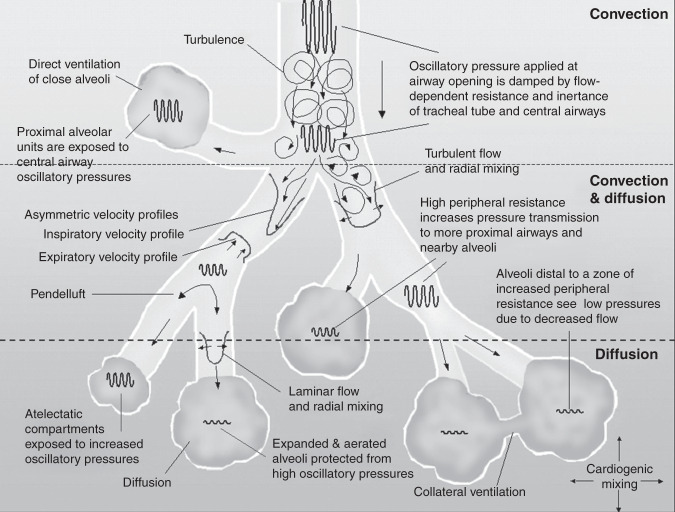
Various mechanisms of gas transport and pressure damping during high-frequency ventilation (HFV). In the trachea and main bronchi, HFV facilitates gas transport by convection, direct ventilation of close alveoli (bulk convection), and asymmetric inspiratory and expiratory velocity profiles. In the smaller bronchi and alveoli pendelluft, cardiogenic mixing, Taylor dispersion with laminar flow, and molecular diffusion are the primary mechanisms. The oscillatory pressure waveform is damped by the inertia of the respiratory system. Atelectatic alveoli are less compliant and are exposed to higher oscillatory pressures compared to normally aerated alveoli. For a detailed review, we recommend “High-frequency oscillatory ventilation: mechanisms of gas exchange and lung mechanics” by JJ Pillow.^15^ Figure 1 was first published by Slutsky A.S. & Drazen J.M. in 2002^17^ and adapted by Pillow J.J.^15^.

During HFV, oxygenation is controlled by the continuous distending pressure (CDP) and the fraction of inspired oxygen (FiO_2_). Optimizing the end-expiratory lung volume (EELV) results in improved oxygenation by reducing atelectasis and intrapulmonary right-to-left shunts. Superimposed on the CDP, HFV generates oscillating pressure swings, resulting in oscillatory tidal volumes (*V*_O_) at supra-physiological frequencies between 8 and 15 Hz to clear CO_2_. The difference between the peak and trough of the pressure wave is referred to as the oscillatory pressure amplitude (Δ*P*_O_). Usually, the Δ*P*_O_ is dampened when moving from the Y-piece to the alveolar compartment by the inertance of the respiratory system.^14^ The *V*_O_ at the Y-piece is close to or smaller than anatomical dead space (1–3 ml/kg), the volume changes at the alveolar level are substantially diminished compared to *V*_T_ in CMV. Ventilation takes place by oscillation-driven mixing of fresh with residual gas by several physical mechanisms (Fig. 1).^15–17^

Similar to CMV, *V*_O_, and frequency (*f*) are essential determinants for CO_2_ washout. However, the equation describing their contribution to ventilation is approximated by *V*_O_^2^ ∙ *f*, also referred to as the diffusion coefficient of CO_2_ (DCO_2_).^18,19^ In HFOV without volume guarantee (VG), an increase in Δ*P*_O_ results in increased *V*_O_ and thus an increased CO_2_ washout, similar to CMV (see “Ventilator technology and modalities” section). Changes in frequency during HFV also affect the *V*_O_, but in an inverse relation, especially when the inspiratory time (t_*i*_) is not constant. For example, increasing the frequency itself will promote CO_2_ clearance, but the concomitant decrease in *V*_O_ in balance may reduce CO_2_ washout. As the latter effect follows nearly a quadratic function, the overall effect of an increase in frequency may be a decreased CO_2_ clearance.

### Ventilator technology and modalities

The three main ventilator modalities providing HFV: high-frequency oscillatory ventilation (HFOV), high-frequency flow interruption (HFFI), and high-frequency jet ventilation (HFJV), will be discussed in this paragraph. HFOV refers to techniques generating an active biphasic displacement of air during both inspiration and expiration. Initially, pressure oscillations were created with a piston pump or an electromagnetically driven loudspeaker membrane and a constant bias flow in the patient circuit. Membranes can create various waveforms and asymmetric inspiration:expiration (I:E) ratios, while piston pumps are limited to sine waves with an I:E ratio of 1:1. This principle of oscillation has been modified in contemporary hybrid ventilators that create full active expiration by using opposite in- and expiratory flows or positive and negative pressure sources.

HFFI uses short bursts of gas delivered directly into the ventilatory circuit, for example, by fast solenoid valves. Expiration is either passive or assisted by a venturi system, which assists the return of pressures to expiratory baseline and results in a modest active negative deflection.^20^

HFJV uses rapid pulses of fresh gas produced by a pinch valve and released as a jet, either directly into the upper airway via a special endotracheal tube or into the Y-piece of the circuit.^21,22^ In contrast to HFOV, exhalation is passive, and to minimize the risk of gas trapping lower operating frequencies are often applied. The settings of the conventional ventilator (peak inspiratory pressure, positive end-expiratory pressure, *t*_*i*_, and *f* ), which is used in tandem, contribute to the mean airway pressure comparable to CMV. A majority of the available studies (bench, animal, and clinical) examined HFOV; this imbalance is reflected in the “Clinical use of HFV” and “Clinical trials” sections.

Most modern (hybrid) ventilators provide the option of simultaneous delivery of CMV and HFV, enabling the application of periodic sighs during HFV, which may assist in lung volume recruitment. However, if conventional sighs are necessary to maintain the optimal lung volume during HFV, this indicates that the selected CDP is probably set too low.

Modern hybrid ventilators also provide *V*_O_ measurement at the Y-piece, enabling the calculation of DCO_2_, and allowing for VG during HFV. During VG, the *V*_O_ set by the clinician is stabilized despite varying lung compliance by automatic adjustments of the Δ*P*_O_. Small cross-over studies in preterm infants have shown that adding VG stabilizes the *V*_O_ and DCO_2_, and thereby reduces the rate of hypo- and hypercarbia.^23,24^ Importantly, VG neutralizes changes in *V*_O_ in response to changes in frequency. As a result, the effect of changes in frequency on CO_2_ clearance during HFV with VG becomes similar to CMV.

Recent studies have shown that HFV ventilators do not perform equally, especially flow interrupters vs. membrane oscillators.^25,26^ There are significant differences between the set and delivered Δ*P*_O_ and the delivered *V*_O_ at different frequencies and conditions of the lung, which are relevant to the clinical application of HFV.

Finally, adequate humidification is essential to avoid airway injury due to insufficient gas conditioning at high flow rates, which are often necessary during HFV.^27–31^

### HFV and lung protection

As previously mentioned, HFV has been considered lung-protective due to the small tidal volume delivery, thereby minimizing alveolar overdistension (volutrauma). However, animal studies have indicated that reducing atelectasis during HFV also contributes to lung protection.^32^ If the underlying lung disease is characterized by alveolar collapse resulting in a low EELV, recruitment and subsequent stabilization of collapsed alveoli are essential for maximum lung protection during HFV. This concept is also referred to as the open lung or optimal lung volume ventilation strategy.

**Fig. 2:**
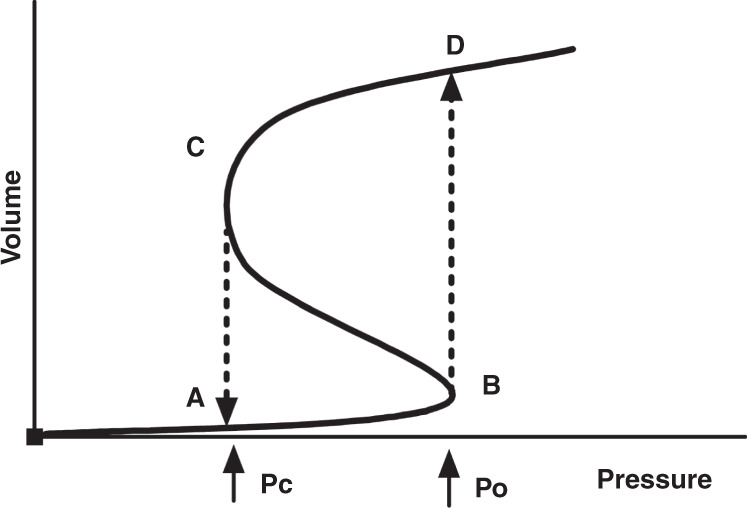
Schematic drawing of the pressure–volume relationship of a single alveolus during inspiration and expiration (solid line). At the start of the inspiration (A), the alveolus is collapsed. At point (B), the pressure increase has reached the critical opening pressure (Po), leading to an immediate volume increase (dashed line) as the alveolus is recruited (D). As the pressure is slowly decreased, there is little volume loss until the critical closing pressure (Pc) is reached at point (C). The alveolus immediately collapses to point (A). Note that Pc is lower than Po due to the law of Laplace.

Figure 2 shows the pressure–volume (*P*/*V*) relationship of an individual alveolus. After a critical opening pressure is reached, the collapsed alveolus opens, immediately resulting in a large volume (and radius) increase (quantal behavior).^33^ As follows by the law of Laplace (${{P}} = \frac{{2 \cdot \gamma }}{r}$), where $\gamma$ is the surface tension and *r* is the radius, the critical closing pressure of the alveolus will be lower than the opening pressure. That relation explains why more volume is maintained at the same airway pressure during deflation compared to inflation.

**Fig. 3:**
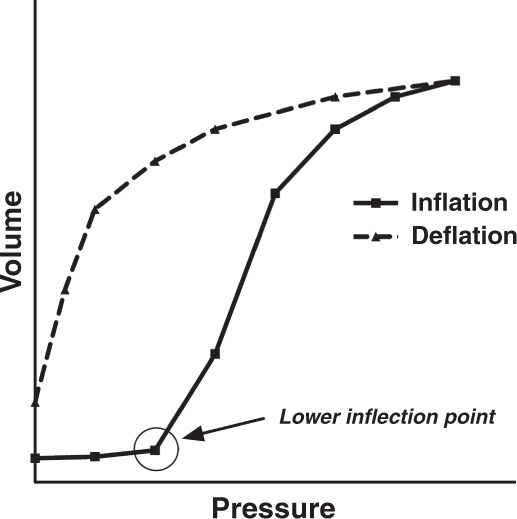
Pressure–volume relationship of the lung showing the inflation (solid line) and the deflation limb (dashed line). Starting from low pressures, the volume starts to rise steeply when in most alveoli Po is reached. As soon as lung volume approaches total lung capacity (TLC), the inflation limb flattens off. Note the apparent difference in lung volume between both limbs at identical pressures (hysteresis).

Figure 3 shows the pressure/volume (*P*/*V*) curve of the lung, which reflects the cumulative volume changes of all alveoli/sacculi in the lung in response to changes in pressure. The inflation limb of the P/V curve shows the changes in lung volume during incremental airway pressures. Usually, it contains a lower inflection point above which lung volume increases linearly. The deflation limb represents the changes in lung volume during decreasing airway pressures starting at total lung capacity. The difference in lung volume between the inflation and deflation limb at a given pressure is referred to as lung hysteresis.^34,35^ Both mathematical models and animal experiments have shown that placing ventilation on the deflation limb of the *P*/*V* curve improves compliance and reduces VILI compared to ventilation on or close to the inflation limb of the *P*/*V* curve.^36–38^

Lung recruitment will optimize EELV and reduce intrapulmonary right-to-left shunt, i.e., perfusion of non-aerated lung units, resulting in improved oxygenation. In homogeneous lung conditions and total resolution of atelectasis, adequate oxygenation is maintained while the FiO_2_ approaches room air. CO_2_ clearance will differ depending on the position of ventilation on the *P*/*V* curve. HFV for lung recruitment was studied mainly with variable CDP and constant Δ*P*_O;_ only when significant hypo- or hypercapnia occurred Δ*P*_O_ was adapted.^39,40^ At the initial flat part of the inflation limb, compliance will be poor, and the resulting *V*_O_ at a given pressure amplitude will be low. When moving up the inflation limb during lung recruitment, compliance will improve, and carbon dioxide will decrease with increasing *V*_O_. Upon reaching the flat upper part of the inflation limb, PCO_2_ will increase due to a decrease in lung compliance and an increase in alveolar dead space. Moving back on the deflation limb, as pressures are reduced, PCO_2_ will decrease again.^41^

## Clinical use of HFV

In clinical practice, HFV should be tailored to the underlying lung disease. HFV can be used in both recruitable and non-recruitable lung diseases. This distinction is, in our view, essential, as only the former will benefit from a lung recruitment procedure during HFV. Examples of recruitable diseases are respiratory distress syndrome (RDS), pneumonia, and meconium aspiration syndrome. In non-recruitable lung diseases, for example, lung hypoplasia after prolonged rupture of membranes or congenital diaphragmatic hernia, the focus should be on avoiding lung overdistension, which may lead to lung injury and air leak syndromes. Combinations of recruitable and non-recruitable conditions might occur. In infants with hypoplastic lung disease, *V*_T_/kg needs to be reduced in CMV to avoid severe VILI, which may lead to hypercapnia. HFOV, despite using small *V*_O_, often can maintain gas exchange potentially without excessive additional VILI.

HFV can be used as a primary or a rescue mode. When using primary HFV, infants failing non-invasive respiratory support are directly started on HFV. This approach is consistent with the concept that HFV aims to prevent lung injury and is not a modality to resolve lung injury. Rescue HFV is often started once other invasive ventilation modes fail to deliver adequate gas exchange or do so only at the expense of injurious ventilator settings. Herein, HFV is used to correct gas exchange and minimize the progression of lung injury.

### Continuous distending pressure

**Fig. 4:**
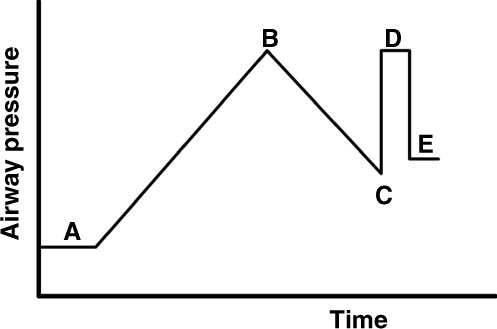
Schematic presentation using oxygenation to optimize lung volume in preterm infants. At the start (A), airway pressure is low, and FiO_2_ is high, indicating a high degree of atelectasis and intrapulmonary shunt. Over time, airway pressures are stepwise increased, resulting in alveolar recruitment, a reduction in intrapulmonary shunt, and an improvement in oxygenation. The latter will allow a stepwise reduction in FiO_2_, thus preventing hyperoxia. Airway pressure is increased until target FiO_2_ indicating optimal recruitment is reached or oxygenation no longer improves (B). The pressure level at point B is called the opening pressure (Po). Airway pressure is stepwise reduced until FiO_2_ starts to increase, indicating alveolar derecruitment (C). This pressure level is called the closing pressure (Pc). After re-opening collapsed alveoli with the known Po (D), airway pressure is set 2 mbar above Pc to ensure the stabilization of lung volume (E).

The primary function of the CDP is to optimize EELV, thereby reducing possible VILI and intrapulmonary shunt, and improving gas exchange. If the patient is suffering from recruitable lung disease, the CDP is also used to reverse atelectasis. One of the crucial challenges in neonatology is monitoring changes in EELV non-invasively and safely at the bedside during lung recruitment. We recommend using oxygenation as an indirect tool to monitor lung recruitment (Fig. 4).^39^ This is based on the assumption that the recruitment of collapsed alveoli will reduce intrapulmonary right-to-left shunt and improve oxygenation. The CDP at the start of lung recruitment depends on whether HFV is used as a primary or rescue mode. When used as a primary mode, the recommended CDP at the start of HFV is around 8 mbar.^39^ If a patient is rescued from CMV failure, most clinicians start with a CDP set 2 mbar above the mean airway pressure used during CMV. Next, the CDP is stepwise increased, and as oxygenation improves, the FiO_2_ is gradually reduced to keep the SpO_2_ within the intended target range. The size of the pressure steps and the time between each step depends on the underlying lung disease.^42^ Monitoring blood pressure during the recruitment procedure is also recommended in order to detect a reduction in cardiac output due to lung overdistension (see also “Complications” section). Other tools like the forced oscillation technique^43^ and electrical impedance tomography^44^ might also be useful in searching for the optimal CDP.

In acute (<72 h old) RDS, we recommend increasing the CDP in 2 mbar steps and waiting at least 3 min between the pressure steps or longer if oxygenation continues to improve.^42^ In neonates >72 h or with more heterogeneous lung disease, bigger steps (3–4 mbar) can be taken while leaving more time (up to 20 min) to assess the effect of each step on EELV.^45^ The CDP is stepwise increased until either the FiO_2_ is reached that defines an optimal EELV or no improvement in oxygenation is seen following 2–3 steps of CDP increase.^39,40^ The CDP at either of these conditions is called the opening CDP (*P*_O_). Once the lung is fully recruited, it is essential to back down with the CDP, moving down on the deflation limb of the pressure–volume curve, taking advantage of the hysteresis of the lung. The CDP is stepwise reduced, keeping the FiO_2_ fixed until oxygenation starts to deteriorate (closing CDP [*P*_C_]). Next, the CDP is increased to the known *P*_O_ and then reduced to 2 mbar above the *P*_C_. This individual recruitment strategy tailors the applied CDP to the severity of (recruitable) lung disease.^39,45,46^

Studies on what FiO_2_ best defines optimal lung recruitment are limited. In the acute phase of RDS, clinicians often aim for a FiO_2_ below 0.30.^39,47^ In the case of more heterogeneous lung disease, somewhat higher FiO_2_ is targeted. It is important to emphasize that the use of oxygenation to guide lung recruitment is hampered in newborns with an extra-pulmonary right-to-left shunt (e.g., persistent pulmonary hypertension of the newborn (PPHN), congenital heart disease). In severe PPHN, the FiO_2_ is not reduced during recruitment, aiming for a high arterial PaO_2_ instead.

Oxygenation-guided lung recruitment requires adequate online monitoring of changes in oxygenation. Possible options are pulse oximetry and transcutaneous PO_2_.

In patients with non-recruitable lung disease, no attempts should be made to increase CDP for lung recruitment. Preferably, the CDP is kept below 10 mbar to avoid the risk of overdistension and subsequent air leaks. Poor oxygenation is primarily caused by extra-pulmonary right-to-left shunt.

In addition to oxygenation, some clinicians assess EELV using chest X-ray. However, studies have shown that the association between EELV based on chest X-ray and a SF6 washout technique is limited.^48^ For this reason, chest X-ray should primarily be used to assess tube position, presence of air leaks or atelectasis, and signs of hyperinflation.^48^

EELV can change during the clinical course. Therefore, a dynamic approach is needed to ensure that the optimal EELV is maintained during the course of lung disease.

### Oscillatory amplitude

At the start of ventilation, a Δ*P*_*O*_ is set that results in slight oscillations of the chest, which usually results in a *V*_O_ around 2 ml/kg at a frequency of 10–12 Hz. Notably, the correlation between the delivered *V*_O_ and the actual PaCO_2_ is limited.^24^ Several conditions will impact the CO_2_ washout at any given Δ*P*_*O*_. First, changes in the underlying lung disease or the position of ventilation on the *P*/*V* curve will influence compliance and thus affect *V*_O_ in HFV without VG. Second, overdistension with an increase in alveolar dead space will decrease CO_2_ clearance. Third, mucus in the airway or position of the tip of the endotracheal tube against the tracheal wall, may dampen the oscillation amplitude and thus result in a rise in PCO_2_.

Nonetheless, HFV is very efficient in removing CO_2_, and this increases the risk of unintended severe hypocapnia, which can compromise cerebral perfusion.^49,50^ Therefore, continuous transcutaneous monitoring of PCO_2_ or DCO_2_ is recommended as a guide for Δ*P*_*O*_ and frequency adjustments.^51,52^ To further reduce the risk of hypocapnia and diaphragmatic dysfunction, we suggest setting the amplitude low enough to allow for spontaneous breathing of the infant.

### Oscillatory frequency

**Fig. 5:**
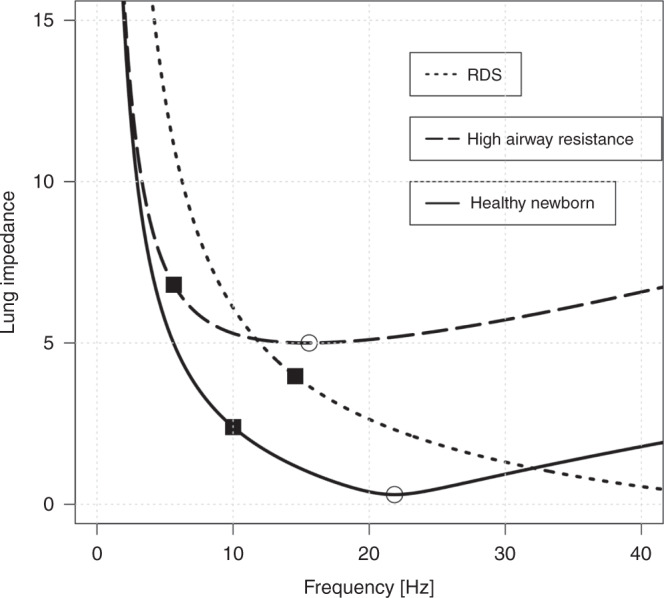
Concept of corner frequency in high-frequency ventilated neonates, according to Dorkin et al.^19,53^ Impedance (*Z*) is plotted against frequency (*f*) following ${|}Z{|} = \sqrt {R^2 + \left( {2\pi \cdot f \cdot I - \frac{1}{{(2\pi \cdot f \cdot C)}}} \right)^2}$ with the specific variables resistance (*R*), compliance (*C*), and inertance (*I*). The solid line represents the impedance/frequency relationship in a healthy lung; the dotted line represents elevated airway resistance; the dashed line reduced compliance. Smaller impedance with increasing frequency represents a lower pressure cost of ventilation and smaller *V*_O_. The resonance frequency (*f*_o_) with the lowest impedance can be calculated by $f_O = \frac{1}{2\pi \sqrt {I \cdot C}}$ and is marked with open rings. The corner frequency (*f*_c_) is inversely related to the time constant of the respiratory system ($f_c = \frac{1}{2\pi \cdot R \cdot C}$), above *f*_c_ (marked with squares) the additional fall in lung impedance is small and the dampening of Δ*P*_*O*_ increases. Thus, low compliance lung disease (e.g., RDS) might benefit from higher frequencies (>10 Hz) and high resistance lung disease from a lower frequency (<10 Hz). However, increasing frequency is limited by decreasing ventilator performance.^57^

Frequencies ranging from 8 to 15 Hz are used in daily clinical practice; however, a set frequency is rarely changed unless the amplitude reaches unconventional settings. The interaction between frequency and ventilation is quite complex; compliance, resistance, and inertance (*I*) determine lung impedance (*Z*) and thereby ventilation at a specific frequency ${|}Z{|} = \sqrt {R^2 + \left( {2\pi \cdot f \cdot I - \frac{1}{{(2\pi \cdot f \cdot C)}}} \right)^2}$.^19,53^ Also, the transmission of the Δ*P*_*O*_ from the airway opening to the peripheral lung units is inversely related to the frequency.^54^ Therefore, using a higher frequency will result in a lower pressure cost of ventilation and smaller *V*_O_, which may reduce the risk of barotrauma.^55,56^ This holds true until the corner frequency is reached above which the additional fall in transmitted Δ*P*_*O*_ is small. The corner frequency is inversely related to the time constant of the respiratory system $({f_{c}} = \frac{1}{2\pi \cdot R \cdot C})$ and might serve as an appropriate target to minimize the pressure cost of HFV (Fig. 5). Low compliance lung disease with a short time constant might benefit from higher frequencies and high resistance lung disease from a lower frequency. However, increasing frequency is limited by decreasing ventilator performance.^57^

### I:E ratio

The I:E ratio can be set by the clinician in most HFV ventilators, and the most widely used settings are 1:1 or 1:2. Using a longer inspiration time will increase *V*_O_ and, thus, a decrease in PCO_2_ unless appropriate reductions are made to the oscillatory amplitude.^58^ Changing the I:E ratio will also affect the transmission of the mean airway pressure from the airway opening to the alveolar compartment. Using a 1:2 ratio will result in a significant drop in mean airway pressure at the alveolar level, especially at higher pressure amplitudes and higher frequencies.^26,32^ This drop is not observed when using a 1:1 ratio, which may lead to gas trapping, which is physically plausible^59^ and was found in animal and lung model studies.^14,54,58,60–62^ An I:E ratio of 2/5 (i.e., 40%) might be a favorable compromise between the risks of air trapping and underinflation.^60^ During expiration, the airways may narrow to the onset of expiratory-flow limitation, and CO_2_ levels reach a plateau and cannot be further reduced. This effect can occur dynamically in some lung areas resulting in hyperinflation (i.e., regional air trapping).^19,61,63,64^

### Weaning and extubation

Similar to CMV, patients on HFV should be weaned to non-invasive support as soon as possible. Anticipating improvement of the pulmonary condition, lowering the CDP (especially at low FiO_2_ requirements) should be attempted at least every 12–24 h. Thereby, optimal EELV is achieved with the lowest possible CDP to avoid progressive hyperinflation leading to lung injury. The Δ*P*_*O*_ is weaned based on the PCO_2_ and the presence of spontaneous breathing efforts. Some clinicians tend to switch back from HFV to CMV for weaning, but extubation directly from HFV is also feasible.^65^ The optimal settings from which to extubate are the subject of ongoing research. For infants with RDS, extubation could be attempted once the CDP reaches 8 mbar and the FiO_2_ is below 0.30.^65^ Infants with more heterogeneous lung disease can probably be extubated from higher settings.

### Complications

HFV has been associated with several complications. Initial trials showed an increase in the risk of intraventricular hemorrhage (IVH), which might have been due to the very effective removal of CO_2_ and lack of adequate PCO_2_ monitoring, but could also have resulted from hampered venous return by higher intra-thoracic pressure,^66,67^ leading to hypocapnia and unstable brain perfusion. More recent studies in extremely preterm infants reported no increase in IVH compared with CMV.^68^ A second reported complication of HFV is an increase in the incidence of air leaks.^68,69^

The application of higher CDP during HFV may also impact hemodynamic stability by impeding venous return. This effect depends on the compliance of the lung and the level of CDP.^70^ Higher CDPs can be applied to low-compliant lungs without compromising hemodynamics. Cohort studies in preterm infants showed that individualized lung recruitment does not significantly impact cardiac output and venous return,^71^ but overdistension reduces right ventricular output.^72^ If a drop in blood pressure does occur during lung recruitment, this should be considered a serious sign of lung overdistension.

## Clinical trials

### HFV in RDS

HFV has mainly been studied in premature infants with RDS. A total of 19 studies, including 4096 infants, have compared primary HFV to CMV.^66,67,69,73–87^ Meta-analyses showed a modest but significant reduction in BPD, death or BPD, and severe retinopathy of prematurity.^68,88^ However, the effect on BPD is weakened by the inconsistency across trials, some showing a benefit of HFV, while more recent trials reported no or small differences between HFV and CMV.^89,90^ Several trials, but not all, also reported an increase in air leaks.^66,68,69,82,87^

**Fig. 6:**
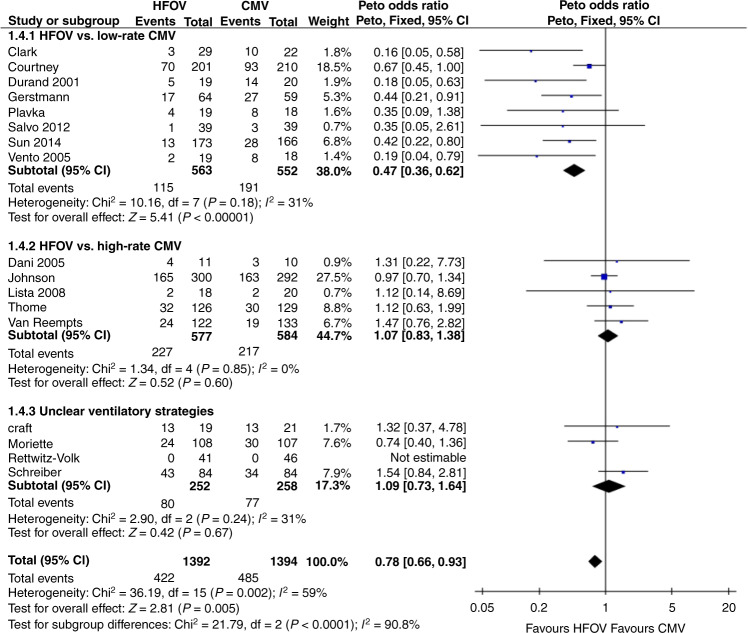
Effect of high-frequency oscillatory ventilation (HFOV) on the rate of BPD at corrected 36 weeks of gestation in preterms with respiratory distress syndrome (RDS). The studies are sub-grouped according to the applied ventilatory strategies as described by Thome et al.,^89^ including the more recent studies.^83–87^ Although an overall significantly lower rate of BPD was observed in the HFOV treated patients, this effect was only evident in studies comparing open lung HFOV strategies to CMV with a respiratory rate of ≤60/min. Comparing HFOV to unregulated CMV or high-rate CMV showed no difference in the pulmonary outcome at 36 weeks. “Events” columns show the number of children with moderate or severe BPD at 36 weeks, and “Total” columns show the number of subjects in the group. Horizontal bars indicate 95% confidence intervals.

Several attempts have been made to explain this heterogeneity across trials, but these are hampered by insufficient data presented in the results section of most trials.^89,90^ Based on the available data, it seems that differences in patient characteristics, supportive therapies, ventilator technology, and the ventilation strategy used during both HFV and CMV are largely responsible for the heterogeneous effect of HFV on BPD.^68^ In some trials, HFV was not combined with a high lung volume strategy and was only applied during part of the ventilation period.^90^ On the other hand, the CMV modes in the available trials differed considerably in the applied frequency and pressure settings. These differences may, at least in part, have compromised the lung-protective potential of both modalities and thus the effect on BPD. Figure 6 shows a forest plot sub-grouped according to ventilation strategies using criteria described by Thome et al.^89^ It seems that using more protective high-rate CMV results in similar BPD rates as HFV. These findings emphasize the importance of the ventilation strategy during both HFV and CMV and the need to optimize the ventilator settings in both modalities.

To date, limited information on the long-term pulmonary outcome is available. In a follow-up study^91^ of the UKOS trial, a significant benefit in adolescent lung function was observed favoring HFOV, even though BPD rates were similar in the HFOV and CMV groups. The HFOV group also had superior results in some cognitive tasks. This study suggests long-term benefits of primary HFV in preterm infants with RDS, regardless of the BPD rating at 36 weeks of gestational age.

### Surfactant application during HFOV

A recently published study investigated the impact of a recruitment procedure applied during HFOV on the efficacy of surfactant treatment via an endotracheal tube. Recruitment with HFOV prior to surfactant administration resulted in a reduced need for mechanical ventilation in the first 72 h of life (primary end-point) but no reduction of BPD.^92^ Several methodological limitations, however, prevent firm conclusions, and it is also unclear how this method compares to avoiding intubation by using the less invasive surfactant application method (LISA).

### HFJV in RDS

To date, four randomized studies comparing HFJV to CMV in preterm infants with RDS are published,^93–96^ of which three are analyzed in detail in a Cochrane review.^97^ Although the results suggest a lower BPD rate in the HFJV groups, drawing conclusions is difficult, as the conventional ventilation protocols were not specified or optimized, and the total number of cases was small. To our knowledge, no studies compare the clinical effects of HFOV to HFJV.^98^

### HFV in other lung diseases

#### Congenital diaphragmatic hernia (CDH)

Aside from retrospective studies and case series documenting the widespread use of HFOV in newborns with CDH,^99–102^ to date the VICI trial is the only prospective randomized trial comparing CMV to HFOV.^103^ This study failed to show a benefit of HFOV over CMV but instead reported a trend to a higher rate of the combined outcome of death or BPD in the HFOV group. This finding might be explained by the fact that lung hypoplasia caused by CDH is a non-recruitable disease, and applying higher CDPs will not offer the same benefits as in recruitable lung diseases.

#### Acute respiratory failure at term

Only a few studies have explored the use of HFV in acute respiratory failure in term infants.^99,104,105^ Most included infants who suffered from meconium aspiration syndrome, respiratory distress, and pneumonia. In ~25–50% of infants with severe respiratory failure, switching from CMV to HFV resulted in a short-term improvement of gas exchange.^106^ Furthermore, combining HFV with inhaled NO provided the highest rate of responders in patients with severe PPHN.^99^ However, these studies were underpowered to evaluate a possible impact on more meaningful clinical outcomes.

#### Air leak syndrome

Although HFV may be associated with an increased risk of air leaks in preterm infants with RDS, some studies have explored the possible benefit of HFV in already established air leak syndrome. Keszler et al.^107^ demonstrated the benefit of HFJV compared to rapid-rate CMV with inspiratory times between 0.20 and 0.35 s. Clark et al.^108^ reported benefits from HFOV in infants with pulmonary interstitial emphysema. Using lower frequencies (5–6 Hz) and an I:E ratio of 1:2 might also be beneficial in established emphysema.^109^

## Current practice and outlook

Currently, only a minority of neonatal units use HFV as the primary mode. More frequently, it is used as rescue ventilation when CMV is failing, or high settings are needed.^13^ However, rescue use of HFV has not been studied sufficiently and should be addressed in future research.

The use of HFV in infants with surfactant deficiency and pulmonary immaturity has been extensively studied. However, its use in other causes of respiratory failure, especially in term newborns, is poorly studied. Future multicenter studies are needed to assess the potential benefit in other conditions that directly or indirectly compromise lung function. Recruitability of the lung, impact on other organs, and hemodynamic compromise must be considered in the study designs.^110^

## Conclusion

HFV is considered a lung-protective ventilation mode, as it uses extremely small tidal volumes to establish an adequate gas exchange. However, animal studies have shown that HFV will only attenuate VILI if combined with an open lung ventilation strategy aiming to optimize EELV. Therefore, HFV is best suited for treating recruitable lung diseases. Among patients with heterogeneous and non-recruitable lung diseases, increasing mean airway pressure may lead to overdistension and pulmonary injury.^111^

Ventilator settings should be individualized based on the pathophysiologic characteristics and severity of the underlying lung disease, aiming to correct lung function with optimized EELV and small *V*_O_. Despite the observed benefits of HFV in animal studies, randomized trials in preterm infants comparing HFV and various CMV strategies have only shown a modest and inconsistent effect on BPD. Long-term data are limited but suggest that additional benefits may be accrued by preterm infants who were initially treated with HFV. Both HFV and CMV remain useful in preterm infant care, as long as lung-protective ventilation strategies are applied. Studies exploring the effect of HFV on other causes of respiratory failure are limited to short-term outcome parameters.
